# Military Ants of the Amazon

**Published:** 1882-02

**Authors:** 


### MILITARY ANTS OF THE AMAZON.

IN *Nineteenth Century* we find the following: The most astonishing insects, if not the most astonishing animals, in the world, are the so-called "foraging," or as they might more appropriately be called, the military ants of the Amazon. They belong to several species of the same genus, and have been carefully watched by Bates, Belt and other naturalists. The following facts must therefore be regarded as fully established.

*Eciton legionis* moves in enormous armies, and everything that these insects do is done with the most perfect instinct of military organization. The army marches in the form of a rather broad and regular column, hundreds of yards in length. The object of the march is to capture and plunder other insects, etc., for food, and as the well organized host advances, its devastating legions set all other terrestrial life at defiance. From the main column there are sent out smaller lateral columns, the composing individuals of which play the part of scouts—branching off in various directions, and searching about with the utmost activity for insects, grubs, etc., over every log and under every fallen leaf. If prey is found in sufficiently small quantities for them to manage alone, it is immediately seized and carried to the main column; but if the amount is too large for the scouts themselves to deal with, messengers are sent back to the main column, whence there is immediately dispatched a detachment large enough to cope with the requirements. Insects or other prey which, when killed, are too large for single ants to carry, are torn in pieces, and the pieces conveyed back to the main army by different individuals. Many insects in trying to escape run up bushes and shrubs, where they are pursued from twig to twig by their remorseless enemies, till on arriving at some terminal ramification they must either submit to immediate capture by their pursuers, or drop down amid the murderous hosts below.

As already stated, all the spoils which are taken by the scouts, or by the detachments sent out in answer to their demands for assistance, are immediately taken back to the main army or column by two smaller columns or carriers, which are constantly running in two double rows (one of each being laden and the other not) on either side of the main column. On either side of the main column there are constantly running up and down a few individuals of smaller size, lighter color, and having larger heads than the other ants. These appear to perform the duty of officers, for they never leave their stations, and while actively running up and down the outsides of the column, they seem intent only on maintaining order in the march, stopping every now and then to touch some member of the rank and file with their antennæ, as if giving directions.

When the scouts discover a wasps' nest in a tree, a strong force is sent out from the main army, the nest is pulled to pieces, and all the larvæ in the nest are carried by the carrier columns to the rear of the army, while the wasps fly around defenseless against the invading multitudes. Or, if the nest of any other species of ant is found, a similarly strong force is sent out, or even the whole army may be deflected toward it, when with the utmost energy the innumerable insects set to work to sink shafts and dig mines till the whole nest is rifled of its contents. In these mining operations the Ecitons work with an extraordinary display of organized co-operation; for those low down in the shafts do not lose time by carrying up the earth which they excavate, but pass on the pellets to those above, and the ants on the surface, when they receive the pellets, carry them only just far enough to insure that they shall not roll back again into the shaft, and, after having deposited them at a safe distance, immediately hurry back for more.

The Ecitons have no fixed nest themselves, but live, as it were, on a perpetual campaign. At night, however, they call a halt, and pitch a camp. For this purpose they usually select a piece of broken ground, in the interstices of which they temporarily store their plunder.

---

Why Cream Rises.—Cream rises best and almost wholly in a falling temperature. The rising is but slow when the temperature ceases to fall. This is because the cream globules are not as good conductors of heat as the water and caseine of the milk. Hence, the latter cools faster than the cream, and its weight is increased. When the temperature becomes equalized throughout, the cream is so very little lighter than the rest of the milk that it rises very slowly. The movement of the cream globules is caused by gravitation, and they rise in the milk just as a balloon rises in the atmosphere. The larger ones rise to the surface first, and the next in size follow, the very smallest coming up last. In some milk there are cream globules so small that they never rise, and they would make very inferior butter if they did, that is, if churned by themselves.

